# IL-22 Production Is Regulated by IL-23 During *Listeria monocytogenes* Infection but Is Not Required for Bacterial Clearance or Tissue Protection

**DOI:** 10.1371/journal.pone.0017171

**Published:** 2011-02-15

**Authors:** Amy C. Graham, Karen D. Carr, Amy N. Sieve, Mohanalaxmi Indramohan, Timothy J. Break, Rance E. Berg

**Affiliations:** Department of Molecular Biology and Immunology, University of North Texas Health Science Center, Fort Worth, Texas, United States of America; Agency for Science, Technology and Research - Singapore Immunology Network, Singapore

## Abstract

*Listeria monocytogenes* (LM) is a gram-positive bacterium that is a common contaminant of processed meats and dairy products. In humans, ingestion of LM can result in intracellular infection of the spleen and liver, which can ultimately lead to septicemia, meningitis, and spontaneous abortion. Interleukin (IL)-23 is a cytokine that regulates innate and adaptive immune responses by inducing the production of IL-17A, IL-17F, and IL-22. We have recently demonstrated that the IL-23/IL-17 axis is required for optimal recruitment of neutrophils to the liver, but not the spleen, during LM infection. Furthermore, these cytokines are required for the clearance of LM during systemic infection. In other infectious models, IL-22 induces the secretion of anti-microbial peptides and protects tissues from damage by preventing apoptosis. However, the role of IL-22 has not been thoroughly investigated during LM infection. In the present study, we show that LM induces the production of IL-22 in vivo. Interestingly, IL-23 is required for the production of IL-22 during primary, but not secondary, LM infection. Our findings suggest that IL-22 is not required for clearance of LM during primary or secondary infection, using both systemic and mucosal models of infection. IL-22 is also not required for the protection of LM infected spleens and livers from organ damage. Collectively, these data indicate that IL-22 produced during LM infection must play a role other than clearance of LM or protection of tissues from pathogen- or immune-mediated damage.

## Introduction

*Listeria monocytogenes* (LM) is an intracellular, gram-positive bacterium found in soil and water and is a common contaminant of processed meats and dairy products. Ingestion of LM results in translocation of the bacterium through the intestinal epithelial layer. Ultimately, LM disseminates through the blood, infecting the spleen and liver. LM infection can cause septicemia and meningitis in immunocompromised individuals, as well as spontaneous abortions in pregnant women [1]. In the mouse model of mucosal intra-gastric (i.g.) infection, LM is not able to efficiently adhere to the epithelial layer and is thus not able to easily pass through the intestine [1]. This can be overcome by infecting mice through the i.g. route with high doses of LM [2], [3], [4] or using the systemic intravenous (i.v.) route of infection [4]. The systemic model of infection is widely used to study immune responses to LM.

Both innate and adaptive immune responses to LM are important for clearance of the pathogen and for protection against re-exposure. Many cell types, cytokines/chemokines, and effector molecules contribute to these immune responses. Although it is known that the IL-12/IFN-γ pathway is important for activation of macrophages and clearance of LM, the recently discovered IL-23 cytokine pathway has not been extensively studied during LM infection. IL-23, a member of the IL-12 family of cytokines, shares a p40 subunit with IL-12, but is also comprised of a unique p19 subunit [5], [6]. IL-23 is secreted by macrophages and dendritic cells in response to invading pathogens, including LM [7], [8]. Although IL-23 is in the IL-12 family, IL-23 does not have the same functions as IL-12. Instead, IL-23 expands and maintains IL-17 secreting T cells, which are known to produce IL-17A, IL-17F, and IL-22 [9]. IL-23 has been described to play a protective role against extracellular or vacuole-bound pathogens such as *Klebsiella pneumoniae* [10], [11], *Citrobacter rodentium* [12], [13], and *Salmonella enterica* [14], [15], as well as against infections with *Toxoplasma gondii* [16], [17] and *Candida albicans* [18], [19], [20]. We have recently shown that IL-23 is required for the production of IL-17A and IL-17F during LM infection and that the IL-23/IL-17 axis is required for survival and clearance of LM from the spleen and liver [21]. We, and others, have also established that the IL-23/IL-17 axis is required for the optimal recruitment of neutrophils to the liver, but not the spleen, during a primary LM infection [21], [22]. The mechanism by which IL-23 offers protection against LM in the spleen, however, remains unknown.

IL-23 can also directly induce the production of IL-22 from T cells, natural killer (NK) cells, NK T cells, and lymphoid tissue inducer (LTi) cells [13], [23]. IL-22 has the ability to induce the production of antimicrobial peptides [9], [24], [23], [25], as seen in *Citrobacter rodentium* infection [13] and *Klebsiella pneumoniae* infection [26]. These antimicrobial peptides can reduce bacterial burdens and protect the host from death. In addition to inducing the production of antimicrobial peptides, IL-22 can also protect tissues against damage. In the *Klebsiella pneumoniae* model, production of IL-22 was able to protect lung tissue [26]. IL-22 can also protect hepatocytes against acute liver inflammation induced by ConA [27].

A previous publication presented data showing that IL-22 deficient and wild-type C57Bl/6 (B6) mice did not differ in LM burdens in the spleen or liver at day 3 post i.v. infection [27]. However, that study did not investigate the clearance of LM in IL-22 deficient mice during later time-points or during secondary immune responses. Our data using IL-23 deficient mice has shown that increased bacterial burdens are not evident in these mice at early time-points post infection. IL-23 deficient mice only begin to show increased bacterial burdens at day 5 post infection, when compared to B6 mice [21]. These facts would suggest that IL-22 deficient mice might only show increased bacterial burdens at later time-points post LM infection. Furthermore, the IL-23/IL-22 axis has been predominantly shown to provide protection at mucosal surfaces, suggesting that this axis may be important during oral LM infection. The current study investigates the production of IL-22, and the role that IL-22 plays during primary and secondary LM infection, using both systemic and mucosal routes of infection.

## Materials and Methods

### Ethics statement

Animal studies were performed under the approval of the Institutional Animal Care and Use Committee at the University of North Texas Health Science Center. The Office of Laboratory Animal Welfare Assurance Number for The University of North Texas Health Science Center Animal Facility is A3711-01. All efforts were made to minimize suffering to the animals.

### Mice

B6 mice were purchased from Charles Rivers/National Cancer Institute. IL-23p19 knock out (KO) and IL-22 KO mice backcrossed at least 8 times on a B6 background (Taconic) have been previously described [28], [29], and were bred at the University of North Texas Health Science Center. Age and gender matched male or female mice between 5 to 12 weeks of age were used for each experiment. Mice were housed with food and water ad libitum in sterile microisolator cages with sterile bedding at the University of North Texas Health Science Center American Association for the Accreditation of Laboratory Animal Care accredited animal facility.

### *Listeria monocytogenes* infections and quantification of bacterial burden

LM 10403s was grown on brain-heart infusion (BHI) agar plates (BD Bacto) and virulent stocks were maintained by passage through B6 mice. To determine bacterial burdens in mouse intestines during mucosal infection, streptomycin resistant LM (LM/strep^r^) was used. For infection of mice, log-phase cultures of LM or LM/strep^r^ were grown in BHI broth, washed twice, and diluted in PBS to the desired concentration. For primary systemic infections, unless otherwise stated, mice were i.v. injected with ∼1×10^4^ LM via the lateral tail vein. For secondary systemic infections, mice were i.v. infected with ∼1×10^3^ LM, then allowed six weeks to recover and clear the primary infection before being i.v. re-infected with ∼1×10^6^ LM. For primary mucosal infections, unless otherwise stated, mice were i.g. infected with ∼1×10^7^ LM/strep^r^ via the esophageal cavity using a gavage needle. For secondary mucosal infections, mice were i.g. infected with ∼1×10^7^ LM/strep^r^, then i.g. re-infected six weeks later with ∼1×10^8^ LM/strep^r^. To determine LM colony forming units (CFUs), spleens and livers from infected mice were homogenized in sterile double distilled H_2_O. Small intestines from i.g. infected mice were extracted by cutting below the stomach and above the cecum, flushed with PBS to remove debris, and homogenized in sterile double distilled H_2_O. Serial dilutions (1∶10) of the tissues were prepared and 50 µl of each dilution was plated on BHI or BHI/strep agar plates. After overnight incubation at 37°C, colonies were counted, and LM CFUs recovered from each tissue were calculated.

### *In vitro* procedures

Mouse serum was obtained by removing the supernatant from whole blood following centrifugation at 14,000 rpm for 30 min. For experiments using splenocytes for culture or direct ex vivo staining, spleens were homogenized with frosted microscope slides and red blood cells were lysed in Tris-ammonium chloride. Splenocytes were cultured in complete RPMI 1640 medium supplemented with 10% fetal calf serum (Atlanta Biologicals), l-glutamine, vitamins, penicillin/streptomycin, nonessential amino acids, and sodium pyruvate (all supplements from Invitrogen-Gibco). Splenocytes were cultured in the presence of heat-killed LM (HKLM) with a multiplicity of infection of 50∶1 or 10 ng/mL IL-23. Liver leukocytes were prepared as previously described [30]. Isolation of liver leukocytes was performed after resuspending cell pellets obtained from homogenized livers in 35% Percoll medium and layering the cells upon 67.5% Percoll medium. The gradient was centrifuged at 600× g for 20 minutes, and low-density cells were collected from the gradient interface. Cell culture was performed at 37°C in humidified air containing 5% CO_2_.

### IL-22 ELISA

ELISAs were performed on serum and filtered, cell-free splenocyte supernatants. Quantification of IL-22 was performed using antibodies from PeproTech. Cytokine levels were determined by comparison with standard curves generated from recombinant IL-22 (PeproTech) and were analyzed using a Biotek EL808 spectrophotometer.

### Annexin V staining and alanine aminotransferase assays

Splenocytes and liver leukocytes were incubated at 4°C for 15 min with saturating amounts of CD45.2 FITC (BD Pharmingen) and Fc block (BD Pharmingen). Cells were then resuspended in binding buffer (BD Pharmingen), and Annexin V PE (BD Pharmingen) were added. Data was acquired and analyzed within an hour of staining using a Beckman Coulter Cytomics FC500. Serum alanine aminotransferase (ALT) levels were quantified by colorimetric ALT enzyme assays (Biotron Diagnostics Inc.) according to manufacturer's directions.

### Immunohistochemistry and microscopy

Immunohistochemistry was performed as previously described [31]. Briefly, 5-µm sections of frozen spleens and livers from LM infected B6 and IL-22 KO mice were made using a Leica CM 1850 cryostat. Antibody combinations used were purified anti-Ly6G (1A8) (BD Pharmingen) and Difco Listeria O polyserum (Fisher Scientific). Anti-Ly6G antibody was developed with anti-rat Alexafluor 594 (Molecular Probes) and Difco Listeria O polyserum was developed with anti-rabbit Alexafluor 488 (BD Pharmingen). Prolong Gold antifade reagent (Invitrogen) and a cover slip were added to the stained tissues. To view the stained tissue, an Olympus Ax70 fluorescence microscope was used and images were captured with an Olympus DP70 digital camera.

### Statistical analysis

Analyses of variances (ANOVAs) were conducted on the data where appropriate. Bonferroni t-tests and Tukey-Kramer analyses were used for post-hoc analyses. LM CFU data was log transformed prior to analysis, and is represented as such in the figures. Log rank analysis was used to analyze survival curves. A p value of 0.05 or less was considered significant in all cases.

## Results

### LM induces IL-22 production during systemic infection

**Figure 1:**
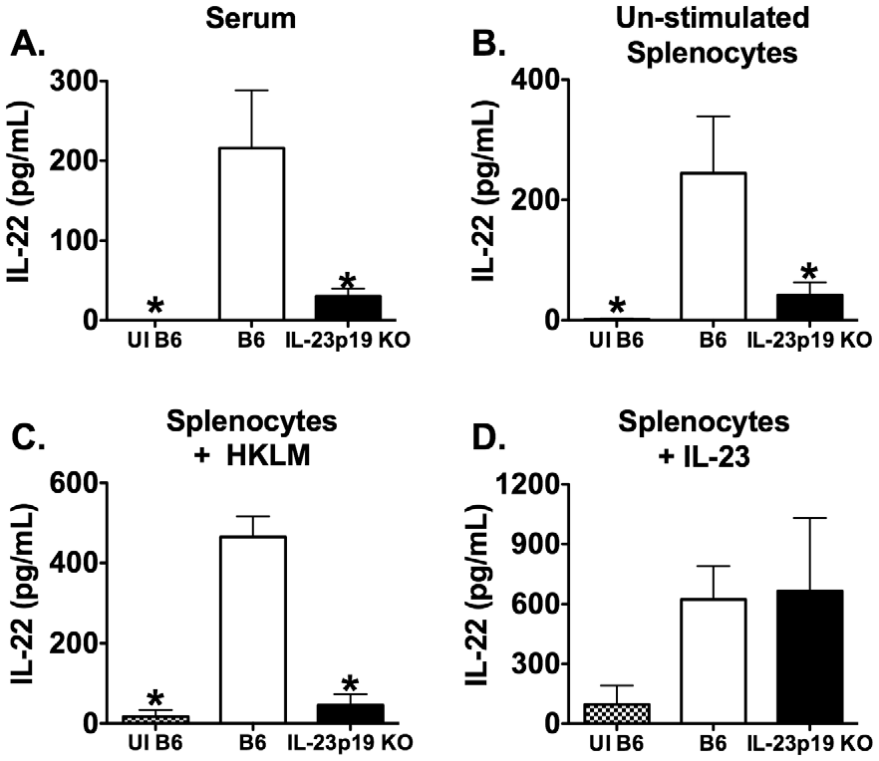
The production of IL-22 during primary systemic LM infection requires IL-23. Serum and spleens were harvested from uninfected B6 mice (UI B6) and B6 and IL-23p19 KO mice i.v. infected with ∼1×10^4^ LM for 3 days. The concentration of IL-22 was measured using ELISA in serum (A) and overnight splenocyte supernatants from un-stimulated (B), HKLM stimulated (C), or IL-23 stimulated (D) cultures. One-way ANOVAs detected significant effects of mouse strain (p≤0.05). An * indicates a significant difference from infected B6 (p≤0.05). These data are representative of two independent experiments. All data are expressed as the mean + SEM (n = 5/group).

IL-22 has previously been shown to play a protective role during certain mucosal bacterial infections [26], [13]. To determine the role of IL-22 during systemic infection, we first sought to determine if IL-22 is produced during LM infection. Serum and spleens were harvested from uninfected (UI) B6 mice or B6 and IL-23p19 KO mice that were i.v. infected with LM for 3 days. The concentration of IL-22 was measured in serum and splenocyte culture supernatants stimulated with or without HKLM or IL-23. LM infection induced IL-22 production in B6 mice in both the serum and the spleen as compared to UI B6 mice. In IL-23p19 KO mice, the amount of IL-22 was reduced in the serum and splenocyte culture supernatants with or without HKLM compared to LM infected B6 mice (**Fig. 1A, B, and C**). Therefore, LM infection induces the production of IL-22, and the production of IL-22 is dependent on IL-23 during a primary systemic i.v. infection. However, there were no differences in IL-22 production from IL-23 stimulated splenocytes from infected B6 and IL-23p19 KO mice (**Fig. 1D**). This suggests that the cells capable of producing IL-22 are still present in IL-23p19 KO mice, but are not able to secrete IL-22 in the absence of IL-23. A similar pattern of IL-22 secretion was observed at days 5 and 7 post infection (p.i.) in serum and splenocyte supernatants, as well as in the liver (data not shown).

### IL-22 is not required for survival during systemic LM infection

**Figure 2:**
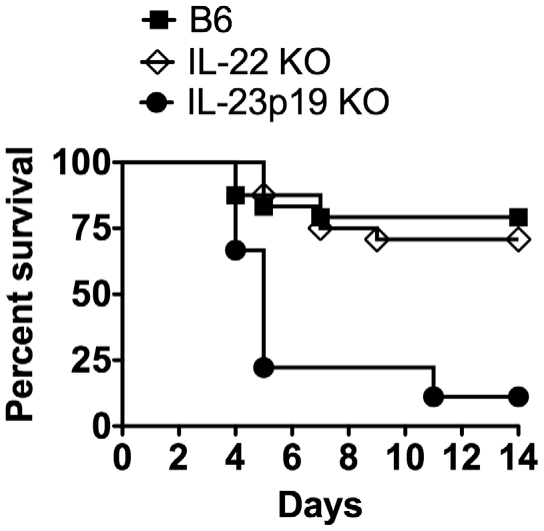
IL-22 is not required for survival during a systemic LM infection. B6, IL-22 KO, and IL-23p19 KO mice were i.v. infected with ∼3.5×10^4^ LM for a survival study. These data are combined from four independent experiments. A log rank analysis detected a significant difference between the survival curves of IL-23p19 KO mice and both B6 and IL-22 KO mice, (p≤0.05), (B6 n = 24; IL-22 KO n = 24; IL-23p19 KO n = 9).

Since IL-22 is being produced during LM infection and is regulated by IL-23, we next wanted to determine if IL-22 has a protective role during a systemic LM infection. To this end, B6, IL-23p19 KO, and IL-22 KO mice were i.v. infected with ∼3.5×10^4^ LM for a survival study. As previously established [21], IL-23p19 KO mice were more susceptible than B6 mice (**Fig. 2**). However, there were no differences in survival between B6 and IL-22 KO mice, and no differences in weight loss (data not shown) during LM infection, suggesting that IL-22 is not required for survival during a systemic i.v. LM infection. To determine if IL-22 was important during a high-dose infection, B6 and IL-22 KO mice were i.v. infected with ∼10^5^ LM. By day 4 p.i., 8/9 B6 and 9/9 IL-22 KO mice had succumbed to the infection. Collectively, these data indicate that although IL-23 is required for protection against i.v. LM infection, IL-22 is dispensable.

### LM induces IL-22 production during mucosal infection

**Figure 3:**
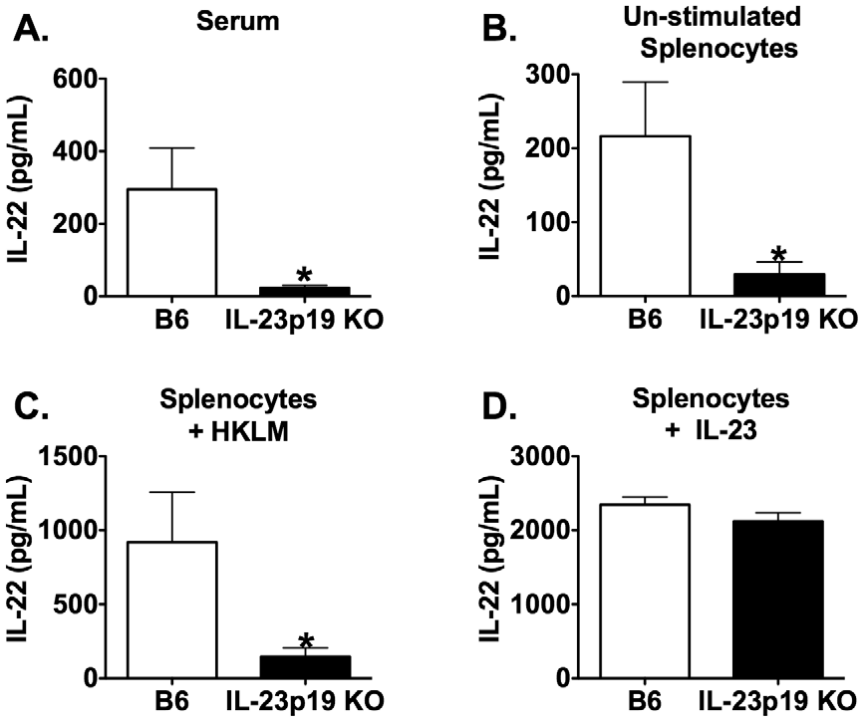
The production of IL-22 during primary mucosal LM infection requires IL-23. Serum and spleens were harvested from B6 and IL-23p19 KO mice i.g. infected with ∼1×10^7^ LM/strep^r^ for 3 days. The concentration of IL-22 was measured using ELISA in serum (A) and overnight splenocyte supernatants from un-stimulated (B), HKLM stimulated (C), or IL-23 stimulated (D) cultures. A two-way ANOVA detected significant effects of mouse strain (p≤0.05). An * indicates a significant difference from B6 (p≤0.05). These data are representative of two independent experiments. All data are expressed as the mean + SEM (n = 4/group).

During the natural route of human infection, LM is ingested from contaminated meats and dairy products. LM is able to move through the intestinal epithelial layer and is transported by the circulating blood to the spleen and liver. To mimic the route of infection in humans, we utilized an oral mucosal model of infection in mice. To determine if IL-22 production requires the presence of IL-23 during mucosal LM infection, serum and spleens were harvested from B6 and IL-23p19 KO mice that were i.g. infected with LM/strep^r^ for 3 days. The concentration of IL-22 was measured in the serum and splenocyte culture supernatants stimulated with or without HKLM or IL-23. In IL-23p19 KO mice, the amount of IL-22 was reduced in the serum and splenocyte culture supernatants with or without HKLM compared to LM infected B6 mice (**Fig. 3A, B, and C**). Therefore, production of IL-22 is dependent on IL-23 during a primary mucosal LM infection. However, there were no differences in IL-22 production in splenocytes re-stimulated with IL-23 (**Fig. 3D**). This again suggests that the cells capable of producing IL-22 are still present in IL-23p19 KO mice, but are not able to secrete IL-22 in the absence of IL-23.

### IL-22 is not required for survival during mucosal LM infection

**Figure 4:**
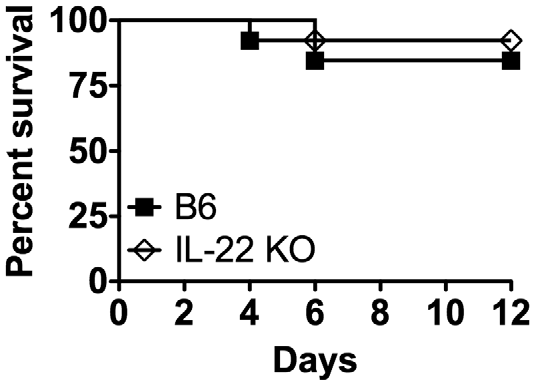
IL-22 is not required for survival during mucosal LM infection. B6 and IL-22 KO mice were i.g. infected with ∼1×10^8^ LM/strep^r^ for a survival study. These data are combined from two independent experiments. A log rank analysis did not detect a significant difference between the survival curves of B6 and IL-22 KO mice, (p>0.05), (n = 13/group).

To discern if IL-22 was required for survival during a mucosal LM infection, mice were i.g. infected with ∼1×10^8^ LM/strep^r^ and observed for 12 days. Weights and clinical signs of illness (posture and condition of fur) of these mice were also observed. There were no differences in survival (**Fig. 4**), weight (data not shown), or clinical signs of illness (data not shown) between B6 and IL-22 KO mice, suggesting that IL-22 production is not required for survival during a primary mucosal LM infection.

### IL-22 is not required for clearance of LM during infection

**Figure 5:**
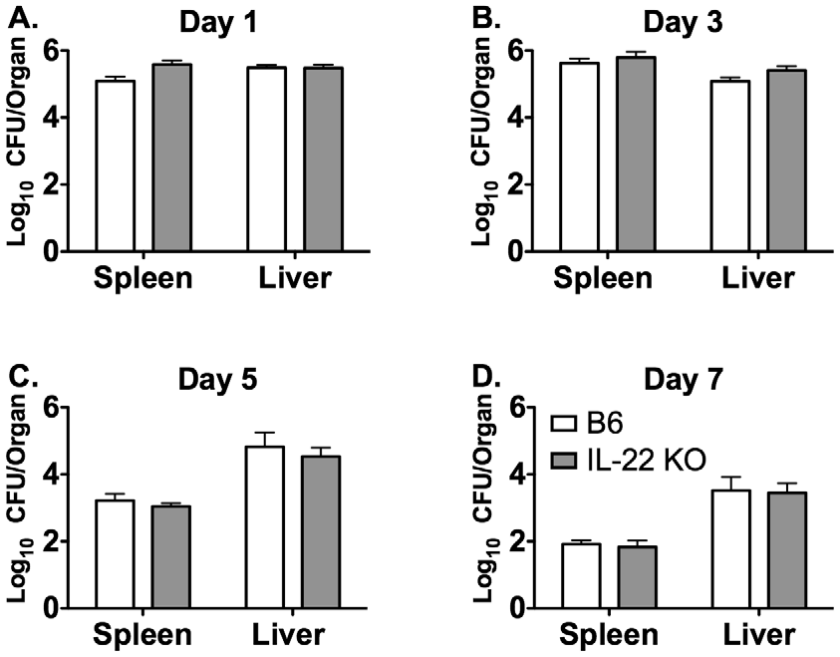
IL-22 is not required for clearance of bacteria from the spleen and liver during a primary systemic LM infection. B6 and IL-22 KO mice were i.v. infected with ∼1×10^4^ LM. Spleens and livers were harvested and bacterial burdens were determined at day 1 (A), day 3 (B), day 5 (C), and day 7 (D) p.i. Two-way ANOVAs did not detect significant effects of mouse strain (p>0.05). These data are combined from two independent experiments. All data are expressed as the mean + SEM (n = 10/group).

Although no differences were observed in overall survival between B6 and IL-22 KO mice, IL-22 could still be impacting the kinetics of clearance of LM from the spleen or liver during infection. To test this possibility, B6 and IL-22 KO mice were infected with LM and bacterial burdens were determined in the spleen and liver. There were no differences in LM CFUs between B6 and IL-22 KO mice at days 1, 3, 5, and 7 p.i. (**Fig. 5A, B, C, and D**). We next wanted to confirm that IL-23, but not IL-22, is required for bacterial clearance from the spleen and liver. To this end, B6, IL-23p19 KO, and IL-22 KO mice were infected with LM and CFUs were determined at day 5 p.i. As previously published [21], IL-23p19 KO mice had higher CFUs than B6 mice. IL-23p19 KO mice also had higher CFUs than IL-22 KO mice, and there was no statistically significant difference between B6 and IL-22 KO mice (**Fig. S1**). These data suggest that IL-22 is not required for clearance of LM from the spleen or liver during a systemic LM infection.

To determine the impact of IL-22 on bacterial clearance during a mucosal LM infection, B6 and IL-22 KO mice were i.g. infected with LM/strep^r^ and spleens, livers, and intestines were harvested at days 1 and 3 p.i. There were no LM CFU differences between B6 and IL-22 KO mice at days 1 and 3 p.i. (data not shown), again suggesting that IL-22 is not required for clearance of LM during mucosal infection.

### IL-22 is not required for protection of tissues during LM infection

**Figure 6:**
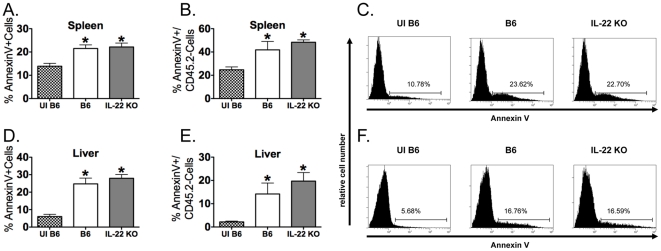
IL-22 does not impact apoptosis in spleens during primary systemic LM infection. B6 and IL-22 KO mice were i.v. infected with ∼1×10^4^ LM for 2 days. Splenocytes and liver leukocytes from uninfected B6 (UI B6), and LM infected B6 and IL-22 KO were harvested and the percentage of apoptotic cells was determined by flow cytometry based on expression of Annexin V. The percentage of apoptosis in all splenocytes (A) and non-hematopoietic, CD45.2-, cells (B) is shown. Representative Annexin V staining of splenocytes is shown in (C). The percentage of apoptosis in all liver leukocytes (D) and non-hematopoietic, CD45.2-, cells (E) is shown. Representative Annexin V staining of liver leukocytes is shown in (F). One-way ANOVAs detected significant effects when comparing uninfected to infected samples (p≤0.05). An * indicates a significant difference from uninfected B6 (p≤0.05). These data are representative of two independent experiments. All data are expressed as the mean + SEM (n = 5/group).

While our data suggest that IL-22 is not required for bacterial clearance during systemic or mucosal LM infection, other models have found that IL-22 protects cells from apoptosis, thereby limiting tissue damage [26], [27], [32], [33]. However, the ability of IL-22 to prevent apoptosis and tissue damage during LM infection is unknown. The peak of apoptosis during LM infection is day 2 p.i. [34], therefore we chose this time-point to investigate the requirement of IL-22 for protection of splenocytes and liver leukocytes from apoptosis. As evident in **Fig. 6A, B and C**, LM infection induced a significant increase in apoptosis in splenocytes. However, there were no differences in the overall percentage of cells undergoing apoptosis in the spleens (**Fig. 6A and C**) of B6 and IL-22 KO mice at day 2 post i.v. infection. In addition, there were no differences in the percentage of non-hematopoietic epithelial cells (CD45.2-) undergoing apoptosis in the spleens of B6 and IL-22 KO mice during LM infection (**Fig. 6B** **)**. Likewise, although LM infection induced an increase in apoptosis in the liver, there were no differences between percentages of apoptotic cells between B6 and IL-22 KO mice (**Fig. 6D, E, and F** **)**. Furthermore, there were no differences in apoptosis between B6 and IL-22 KO mice at days 1, 3, and 5 p.i. (data not shown).

**Figure 7:**
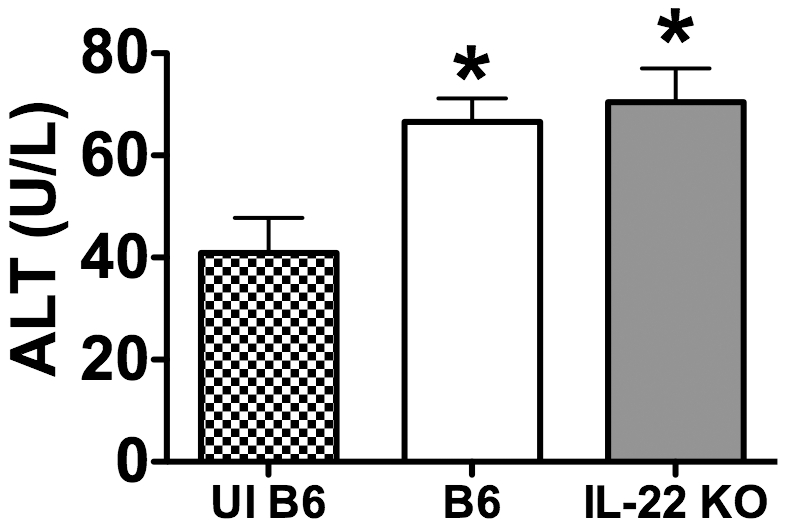
IL-22 is not required for tissue protection in livers during primary systemic LM infection. B6 and IL-22 KO mice were i.v. infected with ∼1×10^4^ LM for 2 days. Serum from uninfected B6 (UI B6), and LM infected B6 and IL-22 KO mice was harvested and analyzed with an ALT detection kit. One-way ANOVAs detected significant effects when comparing uninfected to infected samples (p≤0.05). An * indicates a significant difference from uninfected B6 (p≤0.05). These data are combined from two independent experiments. All data are expressed as the mean + SEM (n = 10/group).

In order to more thoroughly investigate the role that IL-22 might be playing in preventing tissue damage during LM infection, liver damage was assessed by measuring alanine aminotransferase (ALT) in the serum. There were no differences in serum ALT levels between B6 and IL-22 KO mice during systemic LM infection at day 2 p.i. (**Fig. 7** **)**, which is the peak of liver damage during LM infection [35]. This result suggests that IL-22 does not play a role in protecting tissues from damage during systemic LM infection. Similar results were observed at 1, 3, and 5 days post LM infection (data not shown).

Further visualization of LM induced damage in B6 and IL-22 KO mice during systemic infection was accomplished by performing immunohistochemistry on livers obtained from mice infected for 3 days with LM. LM lesions and neutrophils were clearly identified in both B6 and IL-22 KO livers. However, no differences in the number and size of lesions were apparent (data not shown). Collectively, the data from apoptosis assays, ALT assays, and immunohistochemistry suggest that IL-22 is not required for tissue protection during a primary systemic LM infection.

### IL-22 production does not require IL-23 during a secondary LM infection

**Figure 8:**
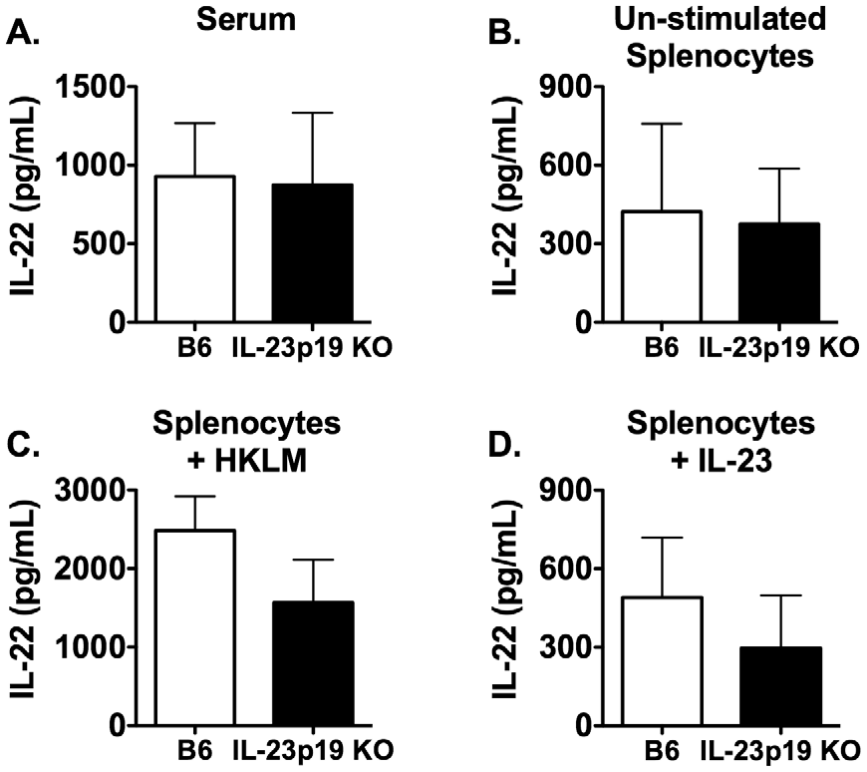
IL-23 is not required for IL-22 production during a secondary systemic LM infection. B6 and IL-23p19 KO were i.v. infected with ∼1×10^3^ LM, then re-infected six-weeks later with ∼1×10^6^ LM. Serum and spleens were harvested 2 days p.i. The concentration of IL-22 was measured using ELISA in serum (A) and overnight splenocyte supernatants from un-stimulated (B), HKLM stimulated (C), or IL-23 stimulated (D) cultures. A two-way ANOVA did not detect significant effects of mouse strain (p>0.05). These data are representative of two independent experiments. All data are expressed as the mean + SEM (n = 4/group).

IL-22 production is dependent on IL-23 during primary systemic and mucosal LM infections (**Fig. 1** **and** **3**). We were interested in determining if IL-22 production was dependent on IL-23 during a secondary systemic i.v. LM infection. To this end, B6 and IL-23p19 KO mice were infected with LM and allowed six weeks to recover. These immunized B6 and IL-23p19 KO mice were then re-infected with LM and spleens were harvested at day 2 p.i. The concentration of IL-22 was measured in the serum and splenocyte culture supernatants stimulated with or without HKLM or IL-23. Unlike what was observed during the primary LM infection (**Fig. 1**), there were no differences between B6 and IL-23p19 KO mice (**Fig. 8A, B, C, and D**). These data suggest that a factor other than IL-23 is able to induce the production of IL-22 during a secondary systemic LM infection.

### IL-22 is not required for clearance of LM or tissue protection during secondary infection

**Figure 9:**
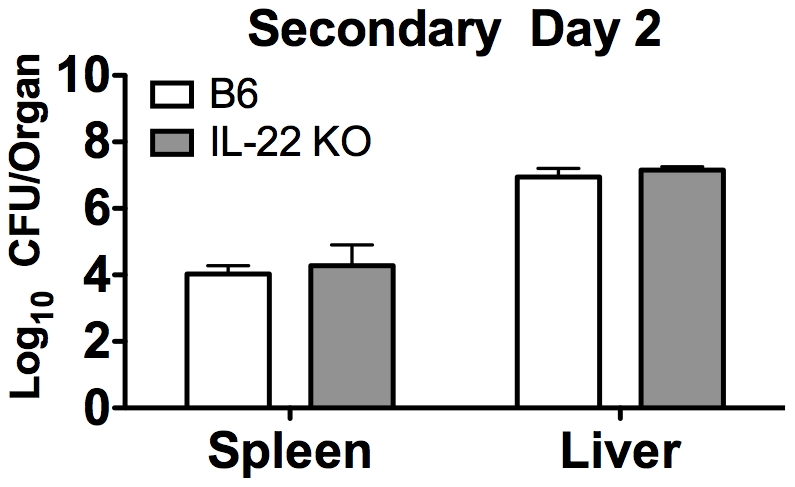
IL-22 is not required for clearance of bacteria from the spleen and liver during a secondary systemic LM infection. B6 and IL-22 KO mice were i.v. infected with ∼1×10^3^ LM, then re-infected six-weeks later with ∼1×10^6^ LM. Spleens and livers were harvested 2 days p.i. and bacterial burdens were determined. A two-way ANOVA did not detect a significant effect of mouse strain (p>0.05). These data are combined from two independent experiments. All data are expressed as the mean + SEM (n = 9/group).

IL-22 is not required for clearance of LM from the spleen or liver during a primary LM infection **(** **Fig. 5** **)**. However, these data do not preclude IL-22 from having a role during a secondary LM infection. To determine if IL-22 impacts the clearance of LM after a secondary exposure to the pathogen, B6 and IL-22 KO mice were re-infected six weeks after a primary LM infection. There were no differences in LM CFUs between B6 and IL-22 KO mice in the spleen or liver at day 2 post secondary infection (**Fig. 9**). To determine the impact of IL-22 on bacterial clearance during a secondary mucosal LM infection, B6 and IL-22 KO mice were re-infected via the mucosal i.g. route of infection six weeks after a primary mucosal LM/strep^r^ infection. There were no differences in LM/strep^r^ CFUs between B6 and IL-22 KO mice in the spleen, liver, or intestine (data not shown).

During a secondary systemic LM infection, there were no differences in the percentage of apoptotic cells in spleens between B6 and IL-22 KO mice (data not shown). Likewise, there were no differences in serum ALT levels between B6 and IL-22 KO mice during secondary LM infection (data not shown), suggesting that IL-22 is not required for limiting liver damage. Collectively, these data suggest that IL-22 is not required for optimal bacterial clearance or tissue protection during secondary LM infection.

## Discussion

To date, the primary role discovered for IL-23 is to maintain lymphocytes that secrete IL-17A, IL-17F, and IL-22 or to directly induce the production IL-22 [9], [6]. IL-22, a downstream cytokine of IL-23, has been previously reported to be able to induce the secretion of antimicrobial peptides [9], [6] as well as protect tissues from damage by preventing apoptosis [26], [27]. IL-22 is induced during certain infectious models including *Salmonella enterica* [14], *Toxoplasma gondii* [36], *Citrobacter rodentium* [13], *Klebsiella pneumoniae* [26], and in an induced model of colitis [37]. We now provide evidence that IL-22 is produced during LM infection. During both a primary systemic and primary mucosal infection, the optimal production of IL-22 during LM infection requires IL-23. When IL-23 was added back into splenocyte cultures from IL-23p19 KO mice, the production of IL-22 was restored, suggesting that the cells that are capable of producing IL-22 are present in IL-23p19 KO mice. This is in contrast to IL-17 secreting lymphocytes during LM infection, which require IL-23 for maintenance and differentiation [21]. Our data suggest that IL-23 is required for the optimal production of IL-22 from splenocytes, but not for the maintenance or differentiation of these cells. At day 2 post-secondary LM infection, IL-22 production is not regulated by IL-23. This suggests IL-22 is not completely dependent on IL-23 for production during secondary exposure to LM, and another factor is able to induce the production of IL-22. Previously published literature suggests that IL-6 or IL-12 may also be able to induce IL-22 secretion [32], [29], [38], [27], [39], providing evidence that IL-22 production is not exclusively associated with the IL-23/IL-17 axis [40].

There were no differences in bacterial clearance between B6 and IL-22 KO mice during primary or secondary systemic or mucosal LM infection. The equivalent bacterial clearance in B6 and IL-22 KO mice during a secondary LM infection implies that IL-22 is not involved in the generation of effector T cells against LM as previously shown [27]. Furthermore, these findings suggest that IL-22 is not required for the optimal generation or maintenance of memory T cells specific for LM. Although IL-22 is known to be able to induce the production of antimicrobial peptides, this potential increased production of antimicrobial peptides may not be required for bacterial clearance during LM infection. Antimicrobial peptides, such as RegIIIγ and β-defensins, are important for LM clearance during mucosal infection [41], [42]. Therefore, an alternative explanation may be that IL-22 is not regulating the production of these antimicrobial peptides. Congruent with our findings that IL-22 does not seem to be playing a role in innate bacterial clearance during LM infection, Zenewicz et al saw no differences in LM burdens between B6 and IL-22 KO mice at day 3 p.i. [27]. Importantly, our data now show that IL-22 is dispensable for clearance of LM at days 5 and 7 p.i., as well as during secondary infection. These results were also obtained using oral infection with LM. Similar results showing that IL-22 does not play a role in pathogen clearance were seen in other infectious models, including parasite infection with *T. gondii* and *Schistosoma mansoni*, bacterial infection with *Mycobacterium avium* and *Mycobacterium tuberculosis* [40], as well as fungal infection with *Candida albicans* [19].

While no differences in bacterial clearance were observed between B6 and IL-22 KO mice, IL-22 has also previously been shown to protect tissues against damage during infection with *Klebsiella pneumoniae* or ConA stimulation [26], [27]. However, we have found that IL-22 is not required for spleen or liver protection during primary or secondary systemic LM infection. A similar result was also seen in the livers of mice infected with *S. mansoni* and *T. gondii*, and in the lungs of mice infected with *M. tuberculosis* and *M. avium* [40]. As mentioned previously, the natural human route of infection for LM is an oral mucosal infection. IL-22 might be playing a role in protecting the intestinal tissue during a mucosal LM infection. During an oral *T. gondii* infection, the intestines of WT mice had more pathology than the intestines of mice treated with anti-IL-22 despite the fact that parasite burdens were the same [40]. Even though there were no differences in LM clearance from the intestine between B6 and IL-22 KO mice during a mucosal oral infection, differences in intestinal tissue damage may still exist. However, the observation of no differences in weight loss between B6 and IL-22 KO mice during mucosal LM infection indicates that IL-22 may not be required to protect intestinal tissue from LM induced damage. Another possibility is that IL-22 is required for an optimal acute phase response during LM infection. It has been previously shown that over-expression of IL-22 can lead to systemic effects in mice that are primarily related to an acute phase response [43]. This possibility during LM infection warrants further investigation. It is also possible that IL-22 plays a novel role during LM infection.

We have previously published that IL-23 is required for clearance of bacteria from the spleen and liver during systemic LM infection. Additionally, we have shown that the IL-23/IL-17 axis has the ability to optimally recruit neutrophils to the liver, but not the spleen, during a primary systemic LM infection [21]. These IL-17 recruited neutrophils are likely to be playing a role in the clearance of LM from the liver (unpublished data). In the current study, production of IL-22 did not influence the clearance of LM from the liver or spleen. Therefore, it is likely that the IL-23/IL-17/neutrophil axis, but not the IL-23/IL-22/antimicrobial peptide axis, is important for LM resistance in the liver [21]. In conclusion, while IL-22 is produced during LM infection and this production is regulated by IL-23, the function of IL-22 currently remains unknown during LM infection.

## Supporting Information

Figure S1 **Unlike IL-23, IL-22 is not required for clearance of bacteria from the spleen and liver during a primary systemic LM infection.** B6, IL-23p19 KO, and IL-22 KO mice were i.v. infected with ∼1×10^4^ LM. Spleens and livers were harvested and bacterial burdens were determined at day 5 p.i. A two-way ANOVA detected a significant effect of mouse strain (p<0.05). An * indicates a significant difference from B6 and IL-22 KO mice (p≤0.05). Data are expressed as the mean + SEM (n = 5/group).
(TIFF)
