# Supplementary figures and images for: IL-22 Production Is Regulated by IL-23 During Listeria monocytogenes Infection but Is Not Required for Bacterial Clearance or Tissue Protection
Source: PLoS One. 2011 Feb 15;6(2):e17171. doi: 10.1371/journal.pone.0017171 (PMC3039664; doi:10.1371/journal.pone.0017171)

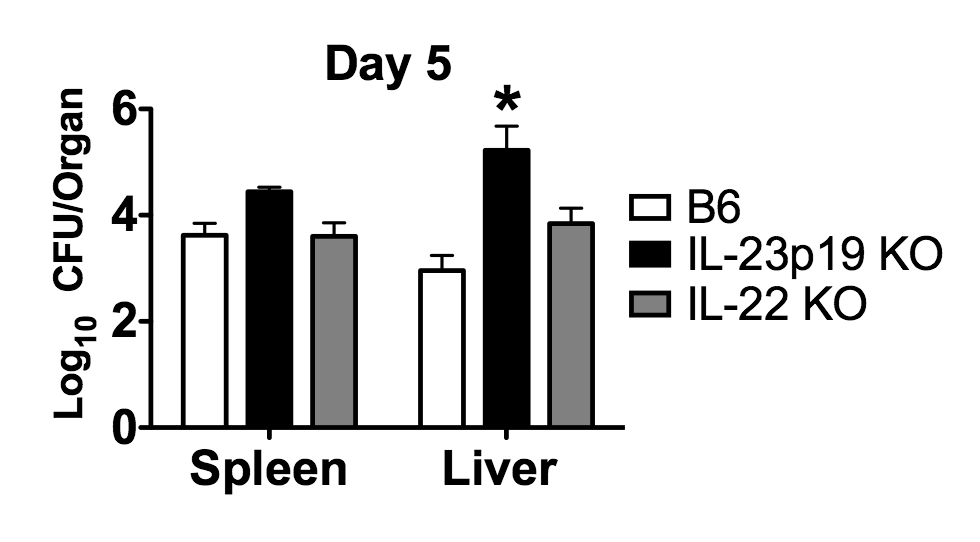

Supplement: Figure S1 — Unlike IL-23, IL-22 is not required for clearance of bacteria from the spleen and liver during a primary systemic LM infection. B6, IL-23p19 KO, and IL-22 KO mice were i.v. infected with ∼1×10^4^ LM. Spleens and livers were harvested and bacterial burdens were determined at day 5 p.i. A two-way ANOVA detected a significant effect of mouse strain (p<0.05). An * indicates a significant difference from B6 and IL-22 KO mice (p≤0.05). Data are expressed as the mean + SEM (n = 5/group). (TIFF) [file pone.0017171.s001.tiff]
